# Reducing the channel diameter of polydimethylsiloxane fluidic chips made by a 3D-printed sacrificial template and their application for flow-injection analysis

**DOI:** 10.1007/s44211-022-00070-1

**Published:** 2022-02-15

**Authors:** Tomohisa Yamashita, Tatsuya Muramoto

**Affiliations:** Toyama Institute of Health, 17-1 Nakataikoyama, Imizu, Toyama 939-0363 Japan

**Keywords:** PDMS, Chip, 3D print, Fused deposition modeling, Flow injection

## Abstract

Fluidic chips have attracted considerable interest in recent years for their potential applications in analytical devices. Previously, we developed a method to fabricate polydimethylsiloxane (PDMS) fluidic chips via templates made using a low-priced commercial Fused Deposition Modeling (FDM) type 3D printer and polymer coatings. However, in general, methods using a template cannot form a flow channel thinner than the template thickness and the width. In this study, the inner wall of a PDMS fluidic chip was coated with PDMS to create a chip with a channel inner diameter smaller than a template. Then, by measuring the flow signal of methyl orange with a single line, the basic properties of the non-coated and coated chip were investigated. As a result, almost the same flow profile was obtained in non-coated and coated chips at the same linear velocity and the same sample injection length. By coating and narrowing the channel width, it is possible to save the amount of sample and carrier solution. Measuring hydrazine in water using a coated chip was also tried. The calibration curve indicated good linearity in the range of 1–6 ppm. However, a concentration point of 7 ppm deviated. The reason for this deviation was presumably due to inadequate mixing of the sample and reagent. By decreasing the flow rate, the calibration curve indicated good linearity in the range of 1–7 ppm.

**Graphical abstract:**

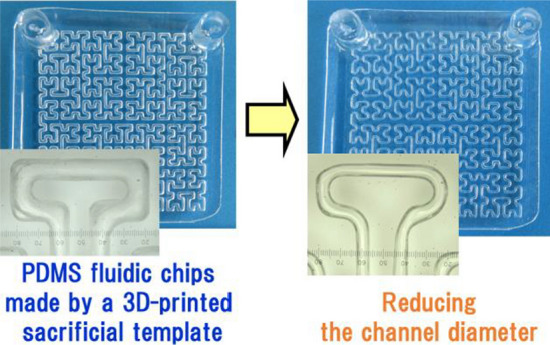

**Supplementary Information:**

The online version contains supplementary material available at 10.1007/s44211-022-00070-1.

## Introduction

Fluidic chips have garnered considerable interest in recent years for their potential application to analytical devices [1–3]. Flow-injection analysis (FIA) is an effective method in analytical chemistry [4–6], and some research on FIA using fluidic chips has been reported [7–9]. One advantage of performing FIA on a fluidic chip is that the flow path can be compacted. The flow path in conventional FIA is composed of tubes; however, bending with a large curvature is difficult, so there is a limit to the densification of the flow path. Conversely, the curvature of the flow path in a fluidic chip can be increased so that a flow path with a complex and dense shape is possible.

Since the FIA flow profile is closely related to the liquid flow, observing liquid flow facilitates FIA research. Fluidic chips can comprise transparent materials, such as glass, PDMS, and resins, thereby allowing the observation of liquid flow in the flow path clearly. On the contrary, in conventional FIA systems, polytetrafluoroethylene tubes or polyetherether ketone tubes, which obstruct the visualization of liquid flow, are generally used for the flow path.

Recently, investigating fabricating fluidic chips using 3D printing has proliferated in many fields [10]. Using 3D printing in the fabrication of fluidic chips has the advantage of enabling the accurate placement of dense channels designed on a computer. PDMS is one of the transparent materials that are often used for fluidic chips [11], but a special 3D printer is required to print this material directly [12]. Therefore, it is common to use a template method when manufacturing a PDMS fluid chip [11, 13, 14]. In a previous study [15], we developed a method to fabricate polydimethylsiloxane (PDMS) fluidic chips via templates made using a low-priced commercial FDM-type 3D printer and polymer coatings, and it was demonstrated that this fluidic chip can be used for FIA. However, in general, the template method cannot form a flow channel thinner than the template thickness and width. In FIA, the amount of the sample and the carrier solution can be saved by reducing the inner diameter of the flow path. Thus, the development of a reduction method is useful. In this study, the inner wall of a PDMS fluid chip was coated with PDMS to form a chip with a channel inner diameter smaller than a template. Then, the flow profile of this improved channel chip was measured.

## Experimental

### Reagents and chemicals

Silpot 184 silicone elastomer was purchased from Dow Corning Toray Co., Ltd. Acetone, ethanol (95), ethanol (99.5), polyethylene glycol 2000 (PEG2000), heptane, hydrochloric acid, sodium hydroxide, methyl orange, *p*-dimethylaminobenzaldehyde, and hydrazine dihydrochloride were obtained from FUJIFILM Wako Pure Chemical Industries (Osaka, Japan). An ABS filament was obtained from WANHAO.

### Apparatus

A Lepton 2 (Magnarecta, Japan) 3D printer was used to fabricate the template. A syringe pump KDS210 (KD Scientific, USA) was used to flow liquids. An SEC2000 (BAS Co., Ltd.) absorption detector was used to measure adsorption. PC-420D (Corning) hotplate was used for heating. An air pump SSPP-2S (Suisaku Corporation) was used to flow air. SPZT50 (Carton) was used as a microscope.

### PDMS chip fabrication

For PDMS chip fabrication, a 3D-printed template composed of ABS was coated with PEG2000. This coated template was immersed in PDMS prepolymer, and subsequently the PDMS prepolymer was cured. Space was created between the ABS template and PDMS by removing the liquid PEG2000 from the channel. A flow path was formed by dissolving the ABS template with a solvent. The PDMS chip fabrication procedure is described in detail in our previous report [15].

### Inner wall coating to reduce channel diameter

Because the PDMS prepolymer is a highly viscous liquid, it is difficult to pour it into the elongated flow path. Therefore, PDMS prepolymer and heptane were mixed in a 1:1 ratio to reduce the viscosity, and the diluted PDMS prepolymer was injected into the channel. Air was injected into the flow channel with an air pump. It was left stationary at room temperature under air flow until the prepolymer in the channel was cured to some extent. This curing time at room temperature took between half a day and 2 days. To cure the prepolymer completely, the chips were placed on a hot plate and heated at 70 °C under flowing air. The inner diameter of the channel was reduced by repeating the prepolymer coating and curing process multiple times. The coating process is illustrated in Fig. S1. Adobe illustrator CS6 was used for analyzing the image.

### Flow-injection measurement system

Liquid flow was controlled with a syringe pump (KD Scientific, USA). Regarding the syringe, all-plastic syringes (HENKE SASS WOLF) were used. SEC2000-DH (BAS Co., Ltd.) was used as the light source. An SEC2000 UV/VIS spectrometer (BAS Co., Ltd.) was used as an absorption detector. A SEC-2F spectroelectrochemical flow cell (BAS Co., Ltd.) was used as the flow cell. To extend the optical path length of the flow cell, the internal gasket was made using PDMS. The internal structure of the flow cell is shown in Fig. S9. A manual injector (7725i, Rheodyne) was used for sample injection. The system was constructed by connecting each instrument and chip with Teflon or polyethylene tubing. Samples were measured without any pretreatment. Microsoft Excel software was used for graph drawing and data integration.

### Measurement of methyl orange

A hydrochloric acid (0.1 M) aqueous solution was used as the carrier solution. As a sample, 1 × 10^−4^ M methyl orange solution was prepared by dissolving in an approximately 1 × 10^−3^ M sodium hydroxide aqueous solution. The detection wavelength was 510 nm. The flow rate and injected sample volume for each chip are shown in Table S1. The sample injection volume was controlled by the volume of the sample loop.

### Measurements of hydrazine

An EtOH-Water mixture (EtOH(99.5):Water = 1:1) was used as the carrier. A reagent solution, 0.12 M *p*-dimethylaminobenzaldehyde (DMAB) in a mixture of EtOH-hydrochloric acid aqueous solution (EtOH(95):concentrated hydrochloric acid = 10:1) was prepared. A flow rate of 0.3 mL/min was used in the both carrier and reagent solution. A hydrazine solution was prepared by dissolving hydrazine dihydrochloride in an approximately 0.12 M hydrochloric acid aqueous solution, and standard solutions were prepared by diluting this hydrazine solution. The injection volume was 0.047 mL, and the detection wavelength was 458 nm.

## Results and discussion

### Reduction of channel diameter

**Fig. 1:**
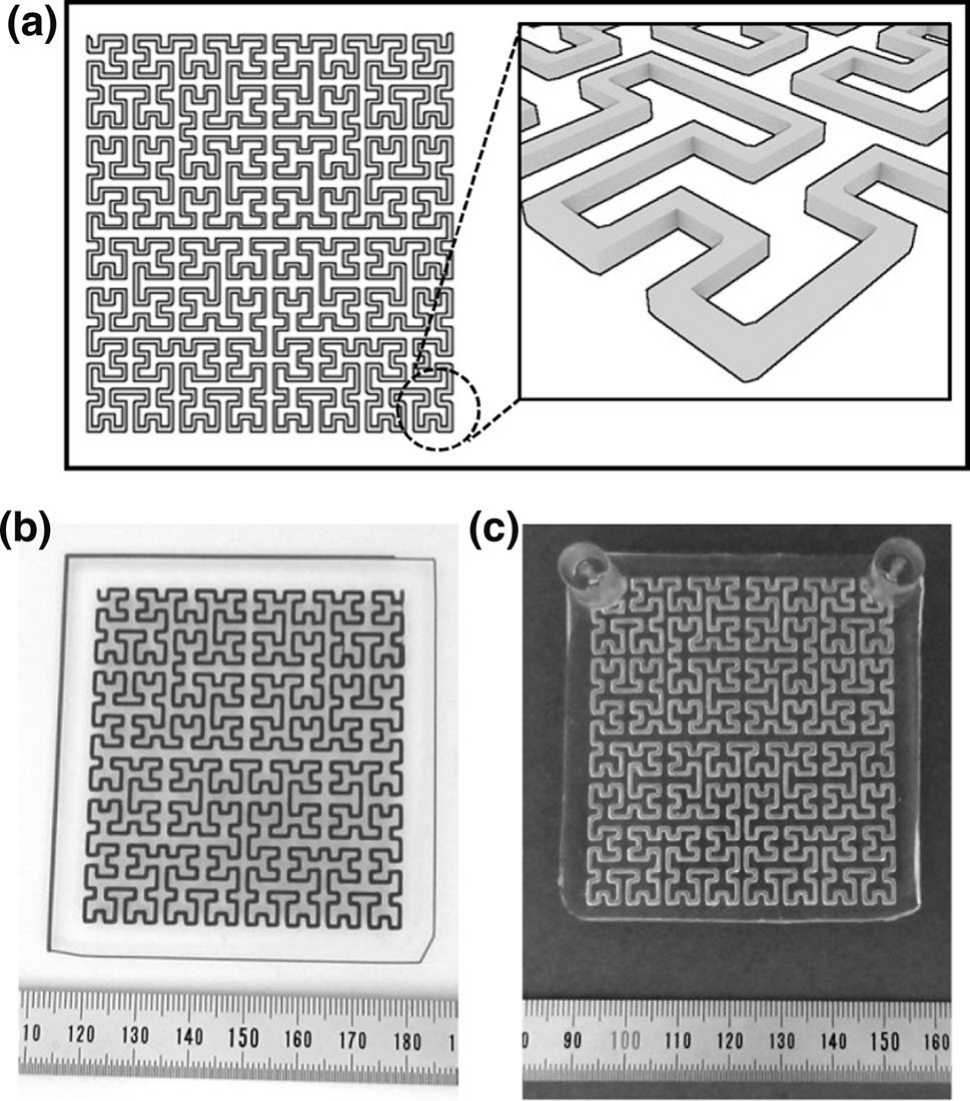
**a** 3D model of a sacrificial template. **b** Image of the ABS template on the print bed after 3D printing. **c** Image of the PDMS chip fabricated by the template method. The contrast and brightness of the pictures were adjusted

Figure 1 shows a 3D model of a template, a 3D-printed template, and a fluidic chip (before the reduction of channel size). The fabrication procedure is described in a previous report [15]. A Hilbert-shaped channel was used to investigate the effect of coatings on structures with many bends.

The thickness and width of the template are related to the diameter of the channel. In this study, since the nozzle diameter of the 3D printer was approximately 0.4 mm, the theoretical minimum printable length in the *x*–*y* direction was considered to be approximately 0.4 mm. We set the height of one layer to 0.3 mm, so 0.3 mm can be considered the minimum printable length in the *z* direction. In general, thinner templates appear to be beneficial in forming narrow channels, but they present some problems; if the template is too thin, it will be difficult to maintain its structure and strength, and it will be difficult to coat it with PEG. Therefore, in this study, the template was printed at a size larger than the theoretical minimum printable length. After forming a large channel, it was gradually narrowed with a coating.

**Fig. 2:**
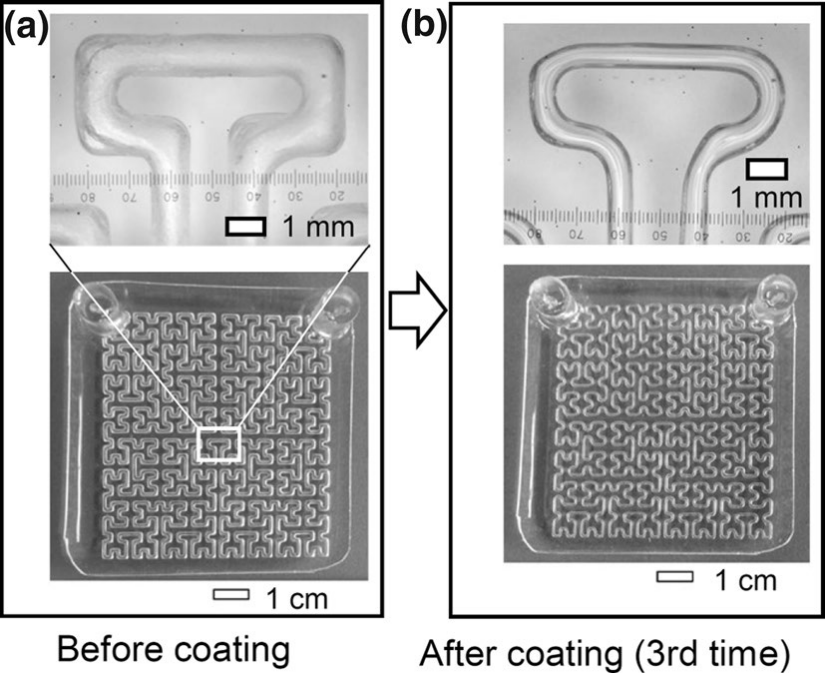
Photographs of the chip **a** before coating and **b** after coating three times. The contrast and brightness of the pictures were adjusted

Figure 2 shows photographs before and after coating the chip three times. Figure S2 shows photographs of the changes in the chip after each coating. Figure S3 shows photographs of top and cross-sectional views of the non-coated and coated channels. During coating of the chips shown in Fig. 2 and Fig. S2, air was flowed from the same inlet three times. As the number of coatings increased, the channel diameter became smaller. In addition, after coating, the rectangular shape of the corner of the flow channel became rounded. It is speculated that the reason for this rounding is the surface tension [16, 17]. The surface tension is a phenomenon in which a liquid forms the smallest possible surface by attracting liquid molecules to each other due to an intermolecular force at the boundary with the air. In this study, the surface tension of PDMS prepolymer and heptane is considered to play an important role in the shape of the coating.

When air was flowed after injecting PDMS prepolymer into the flow path, the PDMS prepolymer applied to the surface was moved to the outlet by air flow. Since liquid PDMS moved with the flow of air, the thickness of the coating was dependent on the location. The coating tended to thicken from the inlet to the outlet. Near to the inlet, the PDMS prepolymer was removed by the flow of air, resulting in a lesser amount of PDMS prepolymer and a thinner coating. Conversely, near the outlet, the PDMS prepolymer continued to be supplied, resulting in a thicker coating. However, a constant channel width is desirable. One way to reduce the difference in chip coating thicknesses was to reverse the direction of air flow as the coating was repeated. Figure S4 shows a photograph after coating three times with and without changing the air flow direction. When the direction of air flow was changed, the variation in coating thickness along the flow path was reduced. The air flow rate during this coating was less than 0.2 L/min.

**Fig. 3:**
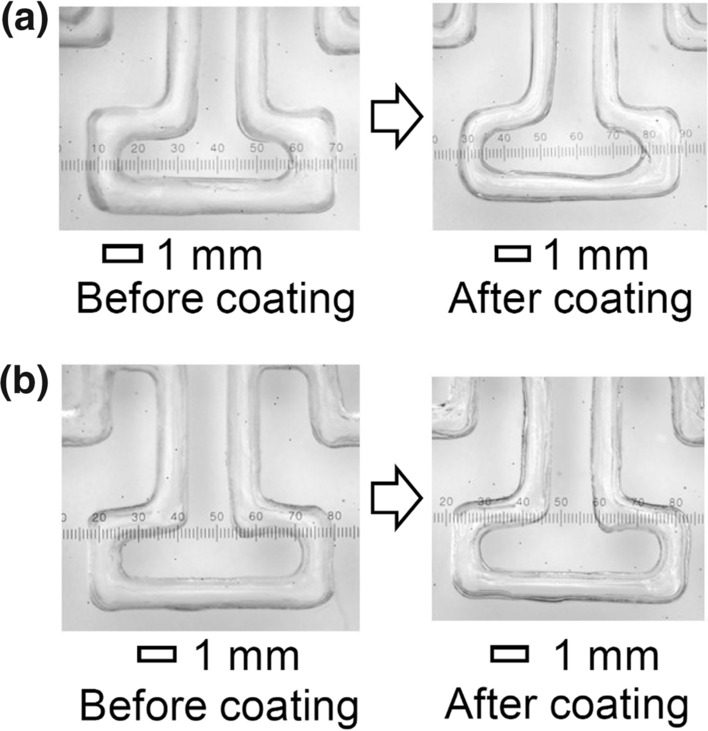
Comparison of the coating thickness when the air flow rate was changed: **a** 33 mL/min and **b** 221 mL/min. The contrast and brightness of the pictures were adjusted

The air flow rate appears to be a factor affecting the thickness of the coating. In this study, the effect of the air flow rate on the coating thickness was investigated. Figure 3 shows a comparison of the coating thickness after one coating under different air flow rates. Detailed results are shown in Fig. S5. The coating tended to become thinner as the air flow rate increased. It is presumed that the coating becomes thinner, because the amount of PDMS prepolymer removed increases with increasing the air flow rate. In this experiment, a chip with a short channel length of approximately 10 cm was used, because the air pump had a limited ability to flow air. In general, the longer is the channel, the larger is the pressure required for permeation. In the case of a chip with a channel length of approximately 2 m, a large amount of pressure is required to pass a high volume of air through the channel. However, the pump used in this study could not apply large pressures. Therefore, it was not possible to flow air at a high rate through a long flow path. To widen the range of the flow rate, the channel length was shortened to reduce the required pressure.

To investigate the reproducibility of the coating thickness, two chips were coated at the same time, and the average channel widths were compared. Calculating the average channel width was performed in the following order: (1) the channel length was measured. The method for measuring the channel length of chip is shown in Fig. S7; (2) the channel volume was measured. The volume of the channel was calculated by measuring the weight of the chip before and after filling the channel with water; (3) the channel cross-sectional area was calculated by dividing the volume by the length; (4) the channel cross-sectional shape was investigated. To calculate the channel width from the cross-sectional area, it is necessary to investigate the cross-sectional shape. In Fig. S3, the cross-section of the channel can be approximated as being circular for both non-coated and coated channels. Hence, the average channel width can be calculated by considering the cross-section of the channel as being circular; and (5) the average channel width was calculated.

The experimental results are shown in Fig. S12. The coating thicknesses were slightly different even under almost the same experimental conditions. The thickness of the coating is considered to depend on the roughness and shape of the wall surface. Therefore, it is likely that differences in the channel shape and wall of each chip caused the difference in thickness of coating.

### Flow-injection measurements

**Fig. 4:**
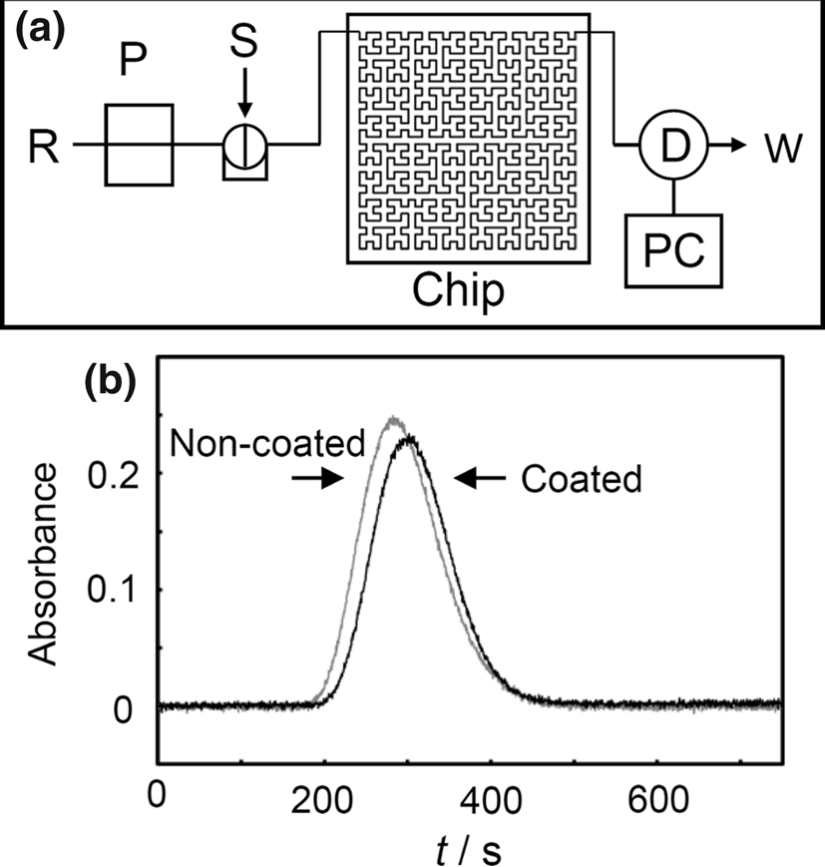
**a** Schematic diagram of the FIA chip system for measurements of methyl orange: R, 0.1 mol/L hydrochloric acid aqueous solution; P, syringe pump; S, sample injector; D, detector; PC, personal computer; W, waste. **b** Comparison of the flow profiles of methyl orange in non-coated and coated chips at the same linear velocity and the same sample injection length. Non-coated chip: flow rate of 0.3 mL/min, injected sample volume of 0.1 mL, average channel width of 0.90 mm (calculated). Coated chip: flow rate of 0.14 mL/min, injected sample volume of 0.047 mL, average channel width of 0.63 mm (calculated)

Experiments were conducted to demonstrate that the PDMS chip, whose flow path is narrowed by coating, is useful for flow-injection experiments. First, by measuring the flow signal of methyl orange with a single line, the basic properties of the non-coated and coated chip were investigated. In this experiment, the measurements were performed under the same linear velocity and the same sample injection length. The parameters of each chip are given in Table S1. A schematic illustration of flow-injection system for methyl orange is shown in Fig. 4a. Figure S6 shows the photos of the flow-injection analysis system and PDMS chips. In this system, it was possible to visually observe a solution of methyl orange flowing through the channel of the chip. The flow profiles measured at the same linear velocity and the same sample injection length are shown in Fig. 4b. As a result, almost the same flow profile was obtained in non-coated and coated chips at the same linear velocity and the same sample injection length. This result indicates that it is possible to save the amount of sample and carrier solution by coating and narrowing the channel width.

Next, a measurement of hydrazine in a water using this chip was tried. Hydrazine is designated by Japan's Ministry of Health, Labor and Welfare as one of the items to be examined in water quality tests as “Items for further study”. Therefore, it is useful to analyze hydrazine for ensuring the safety of water. Furthermore, the analysis of hydrazine is also important for water management in various facilities. It is known that hydrazine has the property of removing dissolved oxygen in water. Thus, hydrazine is often added to water to prevent the corrosion of metal materials [18].

**Scheme 1:**
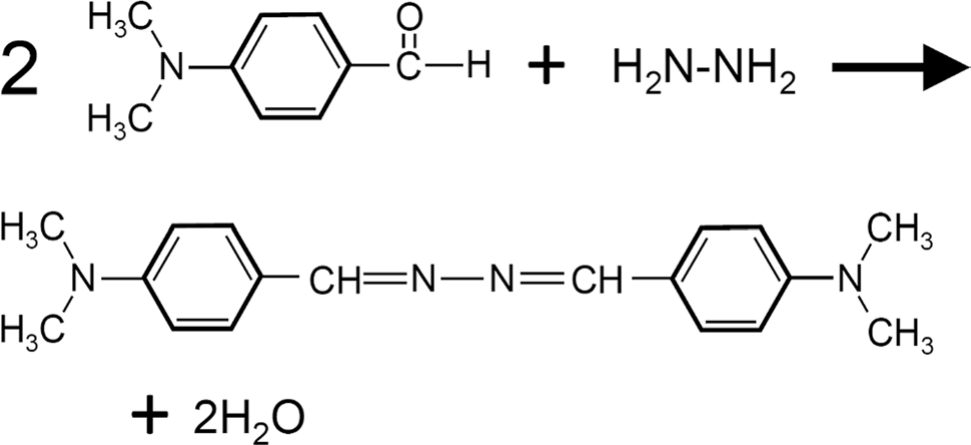
Reaction of DMAB with hydrazine

**Fig. 5:**
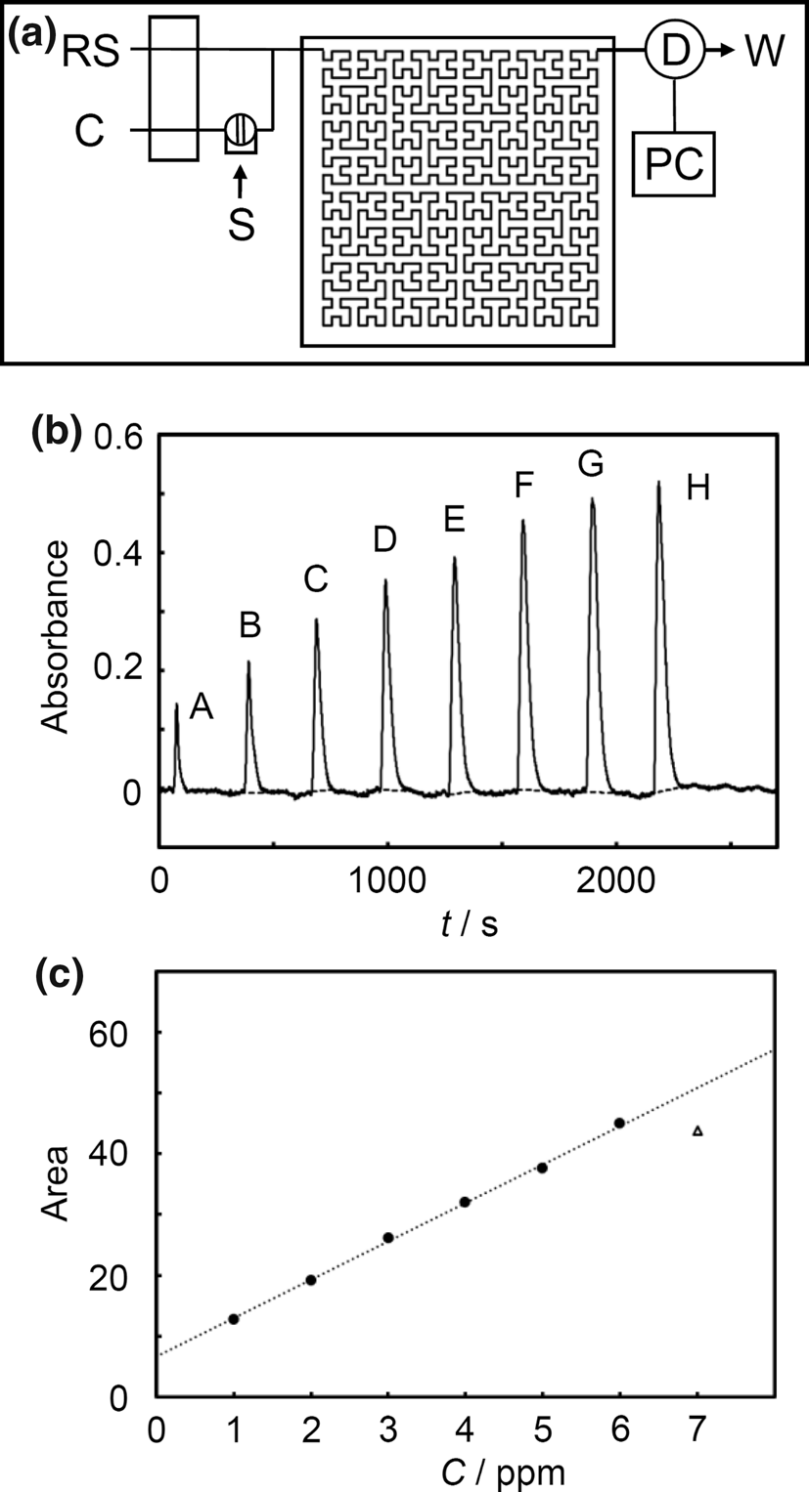
**a** Schematic diagram of the FIA chip system for measuring hydrazine: C, carrier; RS, reagent solution; P, syringe pump; S, sample injector; D, detector; PC, personal computer; W, waste. **b** Flow signal of calibration graph for hydrazine (flow rate of 0.3 ml/min for both carrier and reagent solutions): A, 0 ppm; B, 1 ppm; C, 2 ppm; D, 3 ppm; E, 4 ppm; F, 5 ppm; G, 6 ppm; H, 7 ppm; (The dotted line represents the baseline when the peak is integrated). **c** Calibration curve of the peak area integration obtained by Fig. 5b (7 ppm concentration points are excluded). The dotted line can be represented by the following equation: *y* = 6.3294*x* + 6.5816 (*R*^2^ = 0.9986)

For hydrazine measurements in this study, DMAB was reacted with hydrazine to form a yellow-colored azine complex [19, 20]. The reaction is illustrated in Scheme 1. By measuring the absorption of this azine complex, the concentration of hydrazine can be estimated. A schematic illustration and photo of the flow-injection system used for hydrazine are shown in Fig. 5a and Fig. S8a, respectively. In these measurements, the coated chip shown in Fig. S6c was used. In this system, an EtOH–Water mixture (EtOH(99.5):Water = 1:1) was used as the carrier. When 100% water carriers were used, air bubbles were observed in the flow path, which prevented measurements. The appearance of these bubbles in the fluidic chip could be easily confirmed by visual observation. A photo of air bubbles in the flow path is shown in Fig. S10. To suppress the generation of air bubbles, an EtOH-Water mixture was used as the carrier. One of the advantages of using a transparent PDMS chip is that it enables the direct observation of phenomena in the flow path. Thus, the transparency of the PDMS chip is useful for FIA research.

The flow profiles and calibration curve of peak area integration are shown in Fig. 5b, c, respectively. The calibration curve indicated good linearity in the range of 1–6 ppm. However, the concentration point of 7 ppm was deviated. To investigate the cause, the flow rate of both the carrier and reagent solutions was lowered to 0.14 mL/min for measurements. The flow profiles and calibration curve of the peak area integration are shown in Figs. S11a and S11b, respectively. The calibration curve indicated good linearity in the range of 1–7 ppm. For a flow rate of 0.3 mL/min, it is assumed that the concentration point of 7 ppm was deviated due to insufficient reactions of the sample and the reagent. It is speculated that this deviation can be improved by changing the method of mixing the solutions in the chip.

In Fig. 5b, a peak appears in the flow profile even at 0 ppm. The cause of this peak is considered to be the schlieren phenomenon, which results from an optical inhomogeneity of transparent media. In this study, the carrier is EtOH:water = 1:1, and the solvent of the reagent solution was EtOH:water = 9:1. Therefore, when a standard solution using an aqueous hydrochloric acid solution as a solvent was injected, a peak was observed due to the difference in the refractive indices of EtOH and water. Figure S8b shows the flow profiles when an aqueous hydrochloric acid solution, EtOH, and EtOH:water = 3:1 were injected. When the injected sample was an EtOH or EtOH-water mixture, the difference in refractive index for the carrier was smaller than that for water, and hence the peak was also smaller.

## Conclusions

In previous work, we manufactured a PDMS fluid chip using a 3D-printed ABS template and a polymer coating. However, this method could not form a flow channel thinner than the template. This problem was addressed in this study; we achieved a reduction in the flow path diameter of the PDMS chip by coating the flow path with PDMS. Then, by measuring the flow signal of methyl orange with a single line, the basic properties of the non-coated and coated chip were investigated. As a result, almost the same flow profile was obtained in non-coated and coated chips at the same linear velocity and the same sample injection length. This result indicates that it is possible to save the amount of sample and carrier solution by coating and narrowing the channel width. Next, a measurement of hydrazine in water using this chip was tried. The calibration curve indicated good linearity in the range of 1–6 ppm. However, the concentration point of 7 ppm was deviated. When the flow rate of both the carrier and reagent solutions was lowered to 0.14 mL/min for measurements, the calibration curve indicated good linearity in the range of 1–7 ppm. At a flow rate of 0.3 mL/min, it was assumed that the concentration point of 7 ppm was deviated due to an insufficient reaction of the sample and the reagent. It is speculated that this can be improved by changing the method of mixing the solutions in the chip.

## Supporting information

A schematic illustration of the process of coating a PDMS chip is shown in Fig. S1. Photographs demonstrating the changes in the chip after each coating are shown in Fig. S2. Photographs of the top and cross-sectional views of the non-coated and coated channels are shown in Fig. S3. Photographs after coating three times with and without changing the air flow direction are shown in Fig. S4. A comparison of the coating when the air flow rate was changed is shown in Fig. S5. Photos of the FIA system and the PDMS chips used for the measurements are shown in Fig. S6. The method of measuring the channel length of the chip (Fig. S6b and S6c) is shown in Fig. S7. Photos of the FIA system and the flow profile are shown in Fig. S8. The flow cell and their components are shown in Fig. S9. Photos of air bubbles in the flow path are shown in Fig. S10. The flow signal of the calibration graph for hydrazine (flow rate of 0.14 ml/min for both the carrier and the reagent solutions) and the calibration curve of the peak area integration are shown in Fig. S11. A comparison of the parameters of two chips coated at the same time is shown in Fig. S12. The calculated parameters, flow rate, and injected sample volume for each chip used in Fig. 4b are given in Table S1. This material is available free of charge on the Web at http://www.jsac.or.jp/analsci/.

## Supplementary Information

Below is the link to the electronic supplementary material.

Supplementary file1 (PDF 1064 KB)
