# Supplementary material for: Reducing the channel diameter of polydimethylsiloxane fluidic chips made by a 3D-printed sacrificial template and their application for flow-injection analysis
Source: Anal Sci. 2022 Feb 15;38(3):583–9. doi: 10.1007/s44211-022-00070-1 (PMC8971176; doi:10.1007/s44211-022-00070-1)
Supplement: Supplementary file 1 — Supplementary file1 (PDF 1064 KB) [file 44211_2022_70_MOESM1_ESM.pdf]

Supporting Information

Reducing the channel diameter of polydimethylsiloxane fluidic chips made by a 3D-printed sacrificial template and their application for flow-injection analysis

Tomohisa YAMASHITA*†, and Tatsuya MURAMOTO*

Toyama Institute of Health, 17-1 Nakataikoyama, Imizu, Toyama 939-0363, Japan.

† To whom correspondence should be addressed.
E-mail: tomohisa.yamashita@pref.toyama.lg.jp

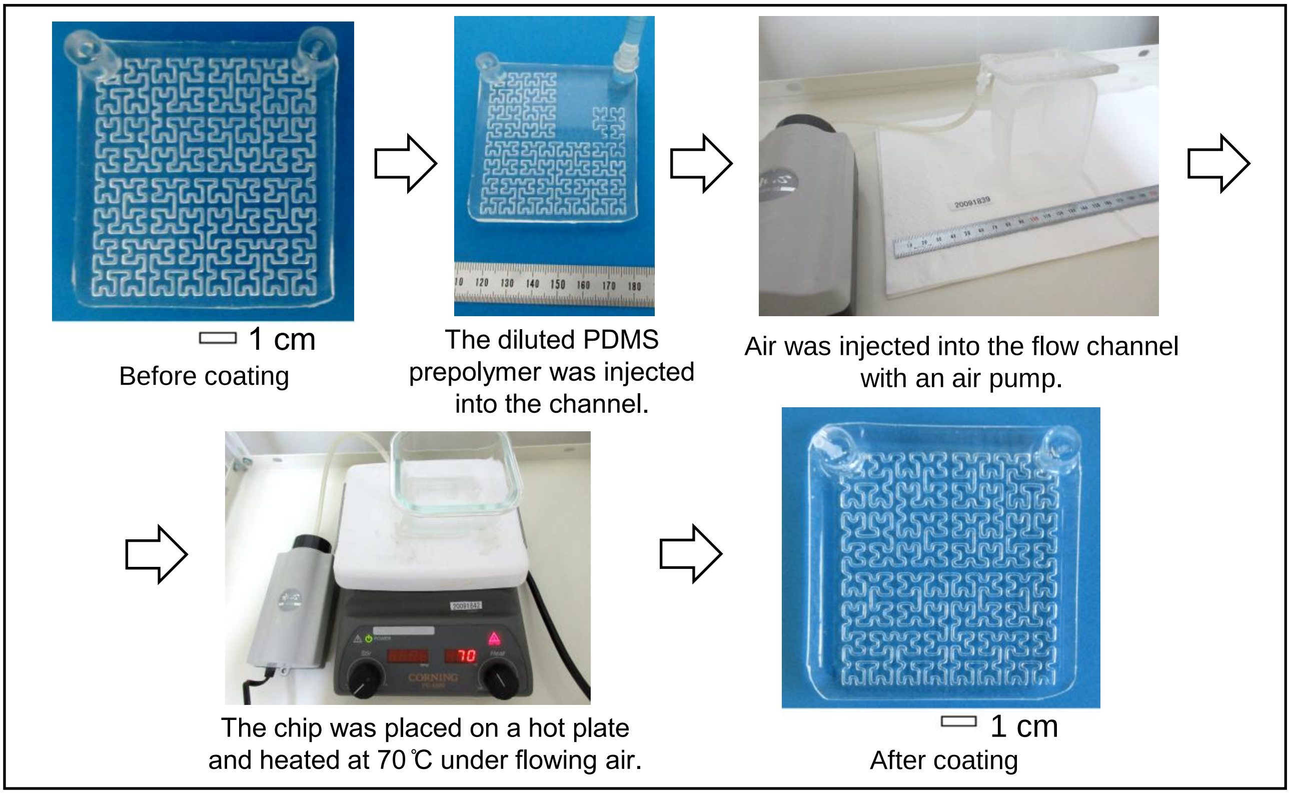

Figure S1 Schematic illustration of the process of coating a PDMS chip.
(Contrast and brightness of pictures were adjusted)

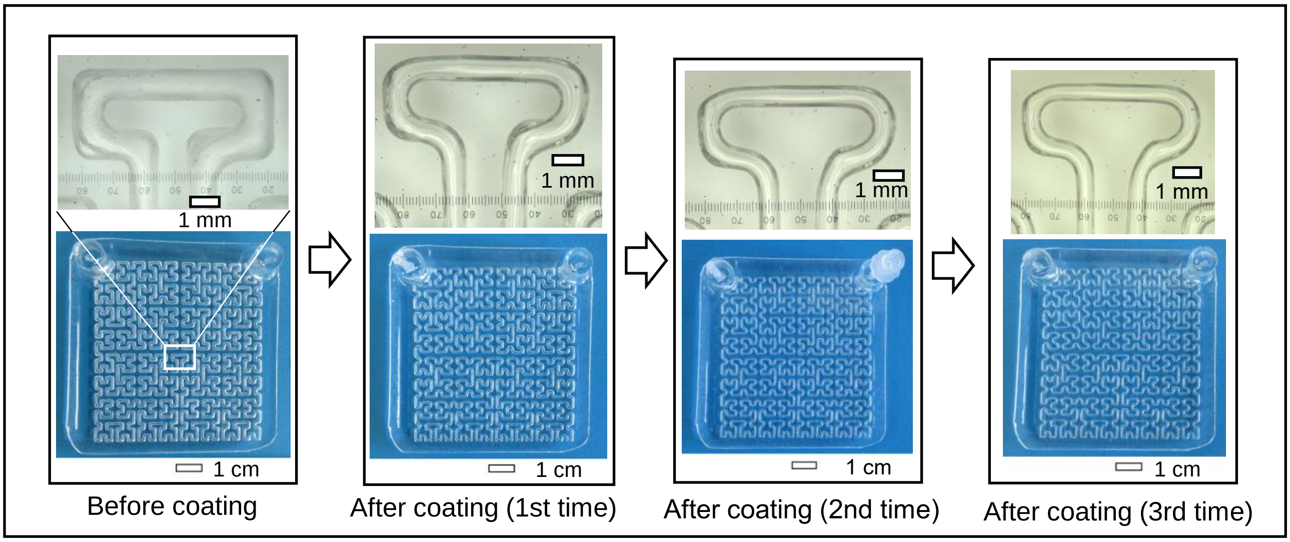

Figure S2 Photographs demonstrating the changes in the chip after each coating.
(Contrast and brightness of pictures were adjusted)

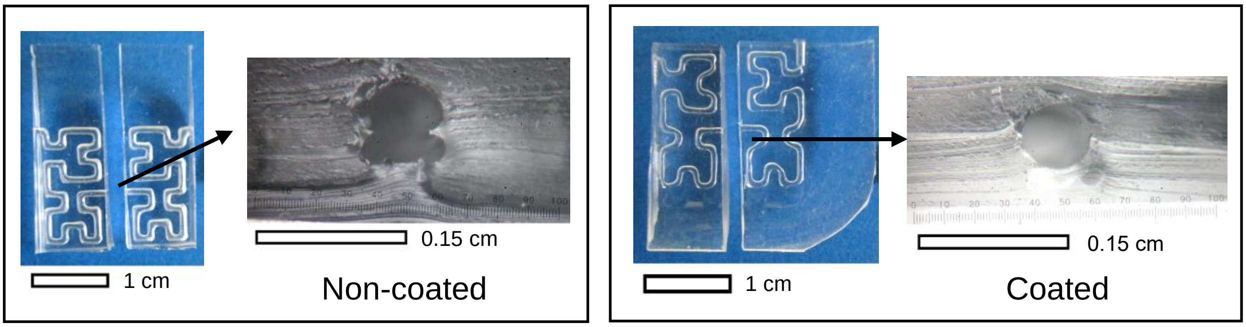

Figure S3 Photographs of top and cross-sectional views of the non-coated and coated channels.
(number of coatings: 3 times) (Contrast and brightness of pictures were adjusted)

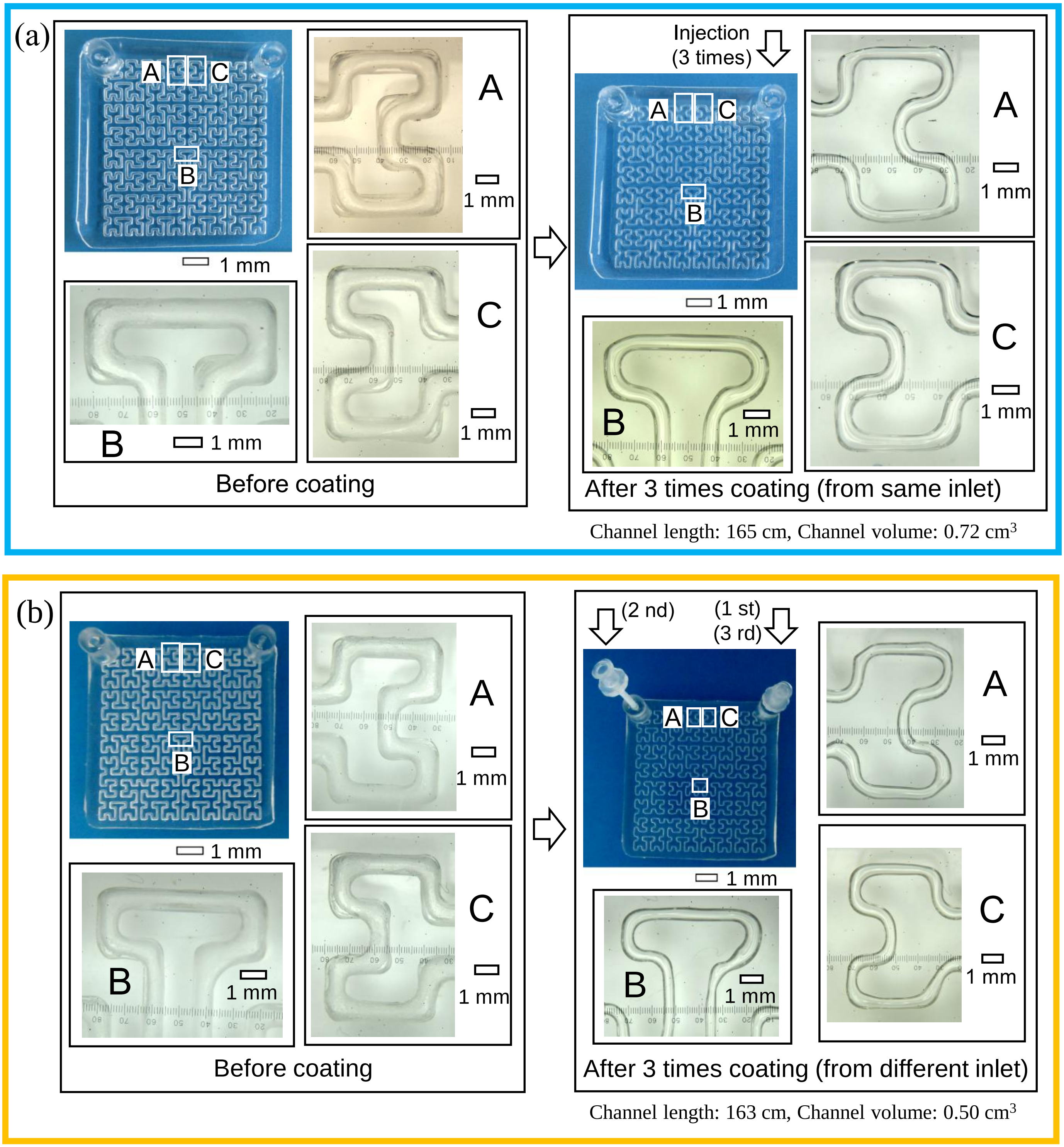

Figure S4 Photographs after coating three times with and without changing the air flow direction.
(a) three times from the same inlet, (b) three times from alternating different inlet.
(Contrast and brightness of pictures were adjusted)

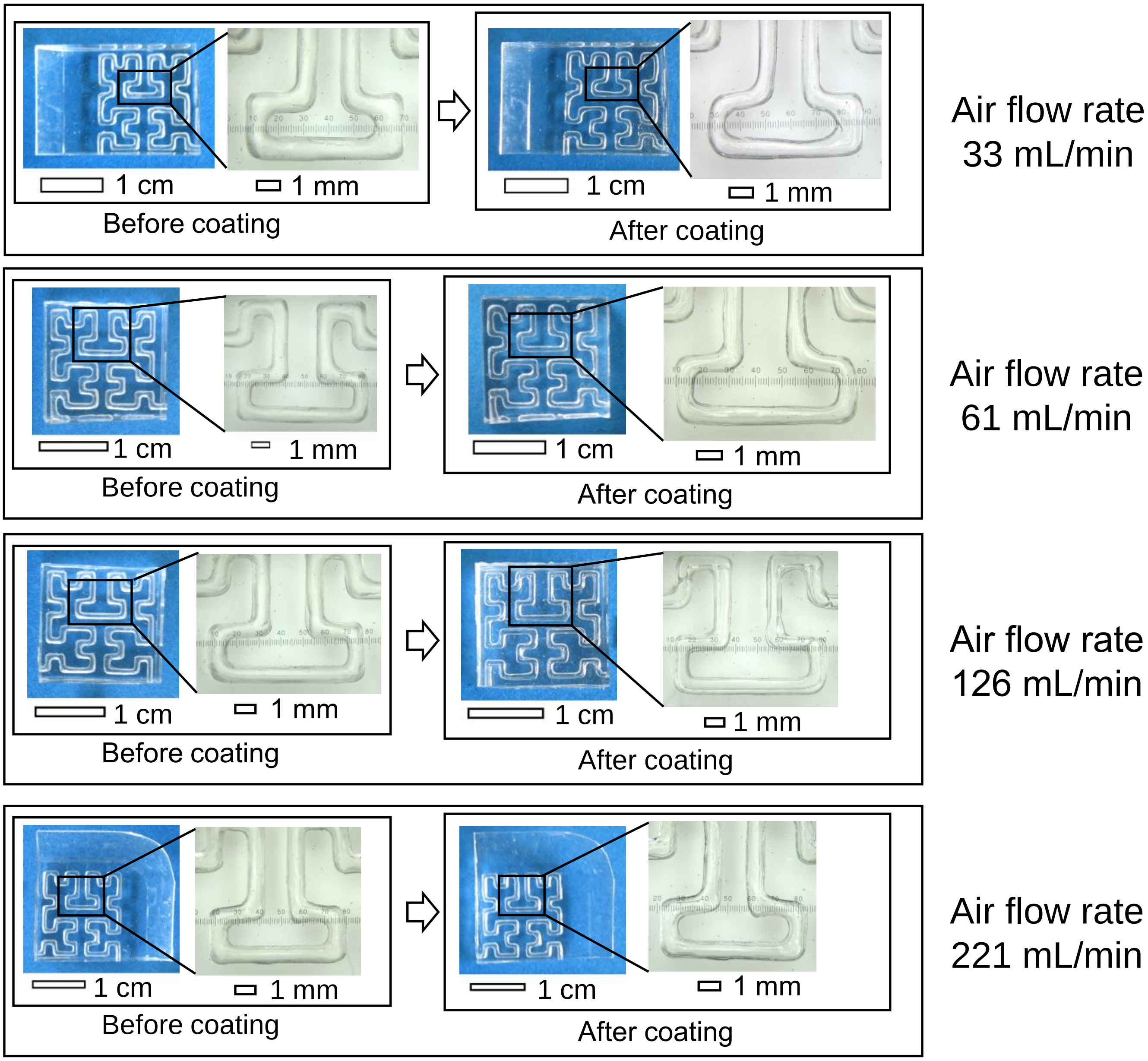

Figure S5 Comparison of coating when the air flow rate is changed.
(number of coatings: 1 time)(Contrast and brightness of pictures were adjusted)

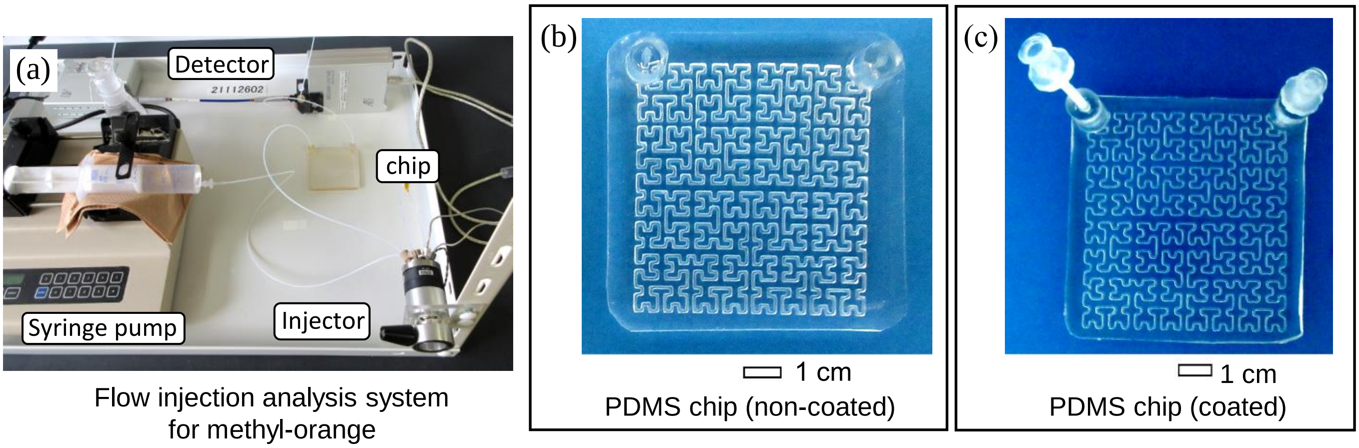

Figure S6 Photos of the FIA system and the PDMS chips used for the measurements.
(a) photo of FIA system for methyl-orange (Injected sample volume: 0.047 mL),
(b) non-coated PDMS chip (calculated average channel width: 0.90 mm) ,
(c) coated PDMS chip (calculated average channel width: 0.63 mm) .
(Contrast and brightness of pictures were adjusted)

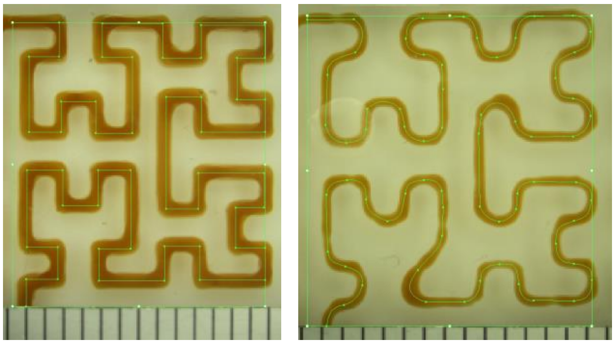

Non-coated channel Coated channel

Procedure

1. Pictures were taken using a microscope.
(In these pictures, colored water was poured into the channel)

2. The photos were opened using Adobe illustrator CS6.

3. The length of the channel was measured by tracing the channel area in the photo with a pass tool.

Figure S7 Method of measuring channel length of chip (Fig. S6b and S6c)
(Contrast and brightness of pictures were adjusted)

	Channel length (cm)	Channel volume (cm^3)	Average cross sectional area (mm^2)	Average channel width (mm)	Flow rate in Fig. 4b (mL/min)	Injected sample volume in Fig. 4b (mL)
Non-coated chip (Fig S6b)	187	1.20	0.64	0.90	0.30	0.10
Coated chip (Fig S6c)	163	0.50	0.31	0.63	0.14	0.047

Table S1 Calculated parameters, flow rate, and injected sample volume for each chip used in Fig. 4b.
（The channel width was calculated by considering the cross section of the channel as circular.）

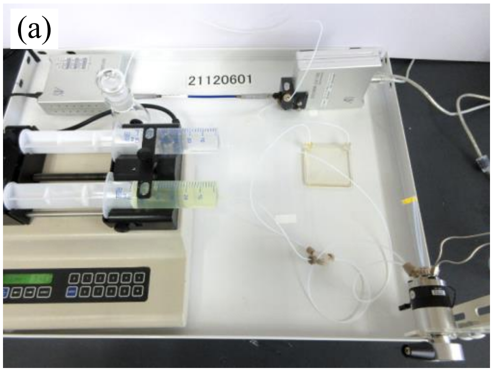

Flow injection analysis system for hydrazine

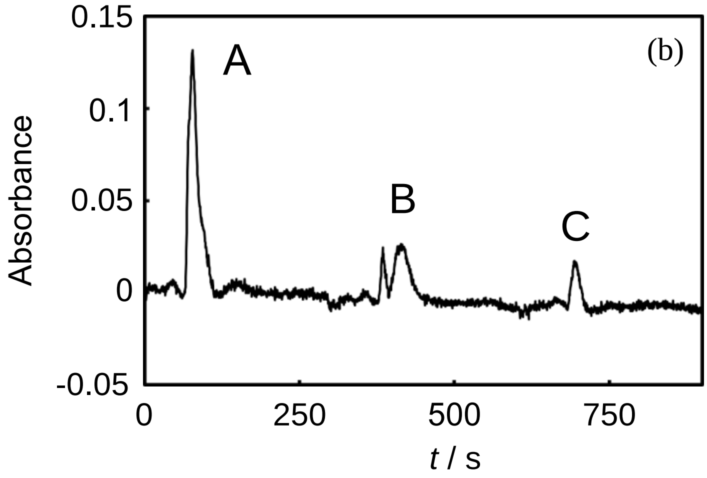

Figure S8 Photos of FIA system and flow profile
(a) FIA system for hydrazine (Contrast and brightness of pictures were adjusted) ,
(b) flow profiles when aqueous hydrochloric acid solution, EtOH, and EtOH:water = 3:1 were injected.
A: aqueous hydrochloric acid solution (0.12 M), B: EtOH, C: EtOH:water = 3: 1.

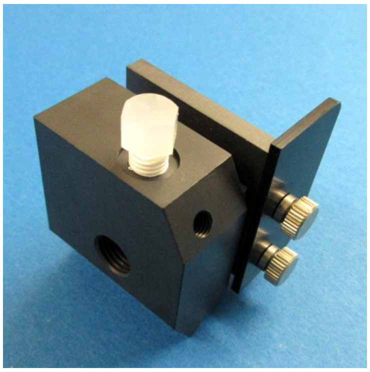

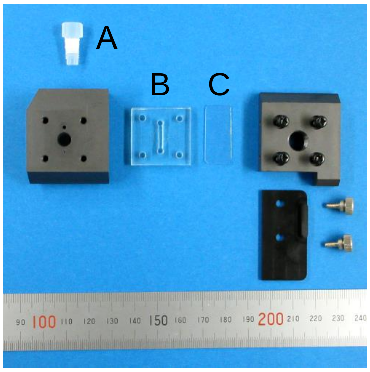

A: Plug
(Teflon tape is wrapped)

B: Gasket
[Material: PDMS
Thickness: 4 mm]

C: Glass plate

Figure S9 Flow cell and their components (Contrast and brightness of pictures were adjusted)

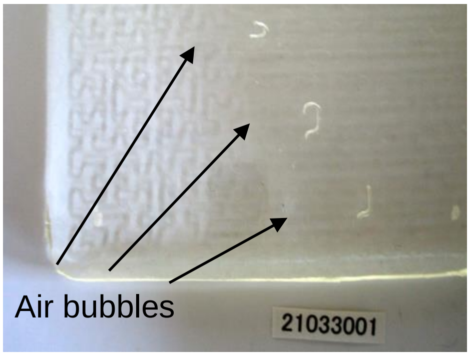

Figure S10 Photos of air bubbles in the flow path.
(Contrast and brightness of pictures were adjusted)

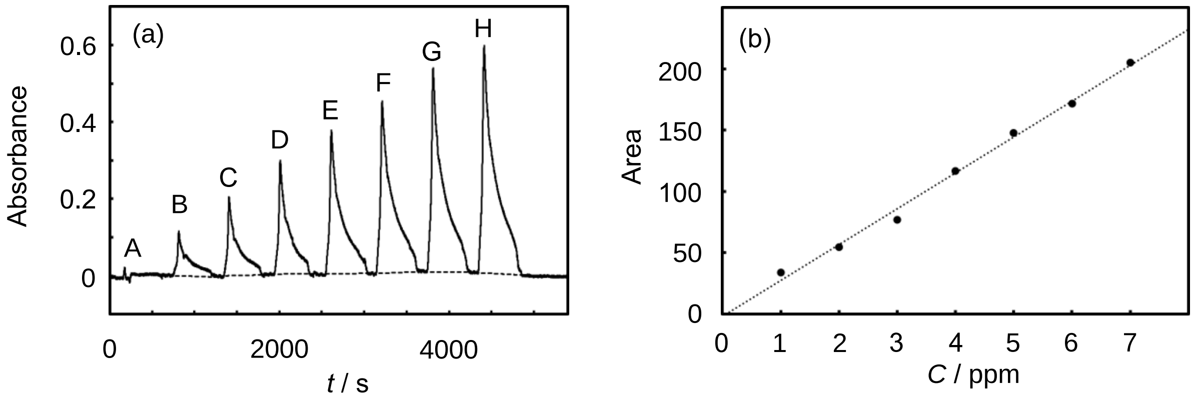

Figure S11 (a) Flow signal of calibration graph for hydrazine (flow rate of 0.14 ml/min for both carrier and reagent solutions): A, 0 ppm; B, 1 ppm; C, 2 ppm; D, 3 ppm; E, 4ppm; F, 5 ppm; G, 6 ppm; H, 7 ppm;
（The dotted line represents the baseline when the peak is integrated）
(b) Calibration curve of peak area integration obtained by Fig. 5b .
The dotted line can be represented by the following equation: y = 29.276x + 1.9798 (R^2 = 0.9938)

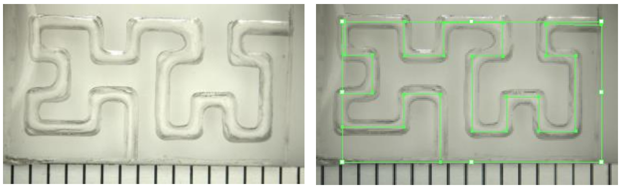

Chip A parameters
Channel length: 60.6 mm, Channel volume: 35.5 mm^3
Average cross sectional area: 0.59 mm^2
Average channel width: 0.87 mm

Before coating

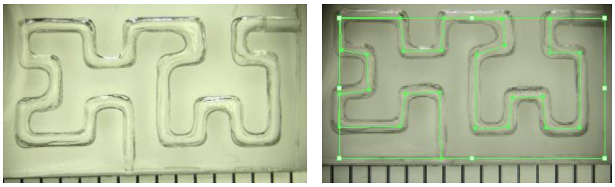

Air flow rate during curing: 48.4 ml/min
Channel length: 56.5 mm, Channel volume: 30.9 mm^3
Average cross sectional area: 0.55 mm^2
Average channel width: 0.84 mm

After coating

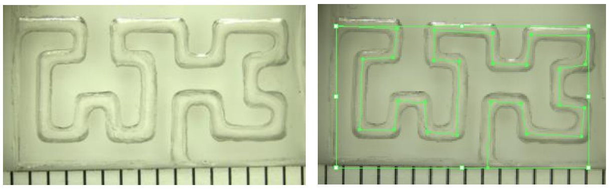

Chip B parameters
Channel length: 58.3 mm, Channel volume: 37.5 mm^3
Average cross sectional area: 0.64 mm^2
Average channel width: 0.90 mm

Before coating

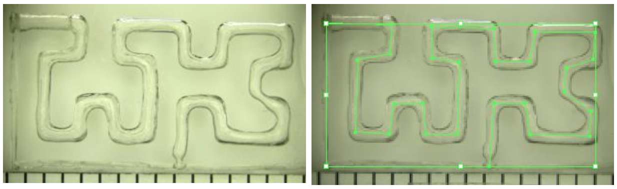

Air flow rate during curing: 49.1 ml/min
Channel length: 55.9 mm, Channel volume: 32.7 mm^3
Average cross sectional area: 0.58 mm^2
Average channel width: 0.86 mm

After coating

Figure S12 Comparison of the parameters of two chips coated at the same time.
(Air flow time: 1 day (room temperature) → half a day (70° C).).
The distance between the scales shown at the bottom of each picture is 1 mm.
(number of coatings: 1 time)(Contrast and brightness of pictures were adjusted)
